# Overexpression of Aquaporin 1 in the Tunica Vaginalis May Contribute to Adult-Onset Primary Hydrocele Testis

**DOI:** 10.1155/2014/202434

**Published:** 2014-04-08

**Authors:** Mami Hattori, Akiko Tonooka, Masayoshi Zaitsu, Koji Mikami, Ayako Suzue-Yanagisawa, Toshimasa Uekusa, Takumi Takeuchi

**Affiliations:** ^1^Department of Urology, Kanto Rosai Hospital, 1-1 Kizukisumiyoshi-cho, Nakahara-ku, Kawasaki 211-8510, Japan; ^2^Department of Pathology, Kanto Rosai Hospital, Kawasaki 211-8510, Japan; ^3^Department of Anesthesiology, Kanto Rosai Hospital, Kawasaki 211-8510, Japan

## Abstract

To investigate the cause of the adult-onset primary noncommunicating hydrocele testis, protein expressions of water channel aquaporins (AQPs) 1 and 3 in the tunica vaginalis were assessed. Frozen tunica vaginalis specimens from patients with adult-onset primary hydrocele testis and control male nonhydrocele patients were subjected to Western blot analysis for the detection of AQP1 and AQP3 proteins. Paraffin-embedded sections of tunica vaginalis specimens were histochemically stained with anti-AQP1 and anti-AQP3 antibodies as well as an anti-podoplanin antibody to stain lymphatic endothelia. Hydrocele fluid was subjected to biochemical analysis. AQP1 protein expression in the tunica vaginalis was significantly higher in patients with adult-onset hydrocele testis than in the controls. The AQP3 protein was not detected in the tunica vaginalis. Histochemically, AQP1 expression in the tunica vaginalis was localized in vascular endothelial and smooth muscle cells. The densities of AQP1-expressing capillaries and lymphatic vessels were similar between the tunica vaginalis of the controls and those of hydrocele patients. Sodium levels were higher in the hydrocele fluid than in the serum. In conclusion, overexpression of the AQP1 protein in individual capillary endothelial cells of the tunica vaginalis may contribute to the development of adult-onset primary noncommunicating hydrocele testis as another aquaporin-related disease.

## 1. Introduction

Hydrocele testis is an accumulation of clear fluid between the tunica vaginalis and testis. Adult-onset primary noncommunicating hydrocele testis causes progressive swelling and local discomfort on the affected side of the scrotum, and this has been attributed to the enhanced secretion and defective absorption of fluid in the space between the tunica vaginalis and testis. The cause is generally unknown. A secondary hydrocele testis is secondary to either inflammation or a neoplasm in the scrotum. The tunica vaginalis in the scrotum is composed of mesothelial cells and submesothelial interstitial tissue, similar to the peritoneum.

Peritoneal dialysis is one of the renal replacement therapies for patients with end-stage renal disease and uses the peritoneum in the abdomen as a dialysis membrane, across which fluids and dissolved substances are exchanged between the blood and dialysate. Theoretical models are used to explain the mechanisms of peritoneal dialysis. The three-pore model of peritoneal transport describes the capillary membrane as a primary barrier that determines the amount of water and solutes transported to the interstitium and peritoneal cavity, mediated by pores of three different sizes [1]. The transport of water and solutes across the capillary, through interstitial tissue, and across the mesothelium was mathematically analyzed in a model [2]. A new version of the model considers the blood flow rate in capillaries and lymphatic absorption from tissue [3].

Water channel aquaporin (AQP) 1 is the molecular counterpart of the ultrasmall pore responsible for transcapillary water permeability during peritoneal dialysis. Water permeability mediated by AQP1 accounts for as much as 50% of ultrafiltration during a hypertonic dwell [4], and the loss of AQP1 can cause ultrafiltration failure in PD patients, together with the formation of a submesothelial fibrotic layer [5, 6].

Aquaporins (AQPs) are a family of water channels that are conserved from lower organisms to mammals. Thirteen mammalian aquaporins (AQP0–AQP12) are known to be widely expressed in various epithelia and endothelia in many organs. AQPs form tetramers in membranes, each individual monomer of which contains six transmembrane *α*-helical domains with the amino and carboxyl termini being located on the cytoplasmic surface of the membrane [7, 8]. The narrow water-permeable pore in each monomer is formed by two highly conserved short hydrophobic portions of amino acid residues called asparagine-proline-alanine (NPA) motifs [9]. Their primary function is to regulate water flow across the plasma membranes of cells. AQPs 3, 7, and 9 transport glycerol as well as water and are called aquaglyceroporins. AQPs 1–4 play significant roles in the mechanism to concentrate urine in the kidney [10]. AQPs also facilitate transepithelial fluid transport in exocrine glands and other secretory epithelia [11].

Human peritoneal tissue was found to express AQPs 1, 3, and 4 in this order, and these may participate in water transport [12]. AQP1 is the most abundant of the AQP family in the mouse peritoneum and the only AQP expressed in the capillary endothelium [5]. Considering the mechanisms of peritoneal dialysis described above, together with the anatomical similarity between the peritoneum and tunica vaginalis, we speculated that hydrocele testis is caused by the overexpression of aquaporin in the tunica vaginalis. We investigated the expression of AQP1 and AQP3 proteins in the tunica vaginalis of patients with adult-onset primary hydrocele testis and demonstrated that AQP1 was overexpressed.

## 2. Materials and Methods

### 2.1. Ethics Statement

This study was conducted in accordance with the Helsinki Declaration after approval by the Ethical Committee of Kanto Rosai Hospital. Written consent was obtained from all participants involved in this study.

### 2.2. Patients

Twenty-one patients with adult-onset primary hydrocele testis (ages 33–82, median: 66) and 19 control male nonhydrocele patients (ages: 22–84, median: 71) in Kanto Rosai Hospital were enrolled in this study. Volumes of hydrocele fluid were available from the charts of 20 patients and were six to 1,600 mL (median: 150 mL). Sixteen control patients had prostate cancer and three had hydrocele funiculi. They had no history of infection, trauma, inguinal hernia, or tumors in the intrascrotal organs. For recent samples, the tunica vaginalis was excised during hydrocele surgery and the hydrocele fluid was collected. The excised tunica vaginalis (*n* = 7) was quickly frozen in liquid nitrogen and then kept at −80°C until being used for Western blotting analysis. As controls, the tunica vaginalis of patients with prostate cancer was excised (*n* = 5) and stored as described above when bilateral orchiectomy was performed as the introduction of endocrine therapy for prostate cancer. When any amount of hydrocele fluid was incidentally found during orchiectomy, the excised specimens were regarded as those of the hydrocele testis. Hydrocele fluid was also subjected to biochemical analysis using TBA-c16000 (Toshiba Medical Systems Corporation, Ohtawara, Japan).

### 2.3. Immunohistochemistry and Morphometry

Paraffin-embedded sections of tunica vaginalis specimens were histochemically stained with an anti-human rabbit polyclonal AQP1 (C-term) antibody (AP23362PU-N, Acris Antibodies GmbH, Herford, Germany), anti-human rabbit polyclonal AQP3 antibody (MBS175443, MyBiosource. Inc., San Diego, CA, USA), and anti-podoplanin antibody (D2-40, Dako, Glostrup, Denmark) for staining lymphatic endothelia as primary antibodies following the manufacturers' instructions using the EnVision System (Dako) and Dako Autostainer (Dako). The expression of AQP1 and AQP3 was determined by a single pathologist (A.T.).

The histology of each section was captured on a Windows computer with a digital camera (DS-Fi1, Nikon Instruments, Japan) at a magnification of 100x, using image-analyzing software (NIS-Elements D, Nikon Instruments, Japan). A scale bar of 100 *μ*m was added to each image for calibration in histological analysis. Thereafter, captured images of sections were analyzed with image-analyzing software (Image J, http://rsbweb.nih.gov/ij/) in order to count the numbers of AQP1-expressing capillaries and podoplanin-expressing lymphatic vessels and measure areas of tunica vaginalis tissues in sections. The numbers of AQP1-expressing capillaries and lymphatic vessels divided by the area were regarded as the AQP1-expressing capillary density and lymphatic vessel density, respectively.

### 2.4. Western Blotting

Frozen tissues were homogenized in ice cold RIPA buffer (25 mM Tris-HCl (pH 7.5), 150 mM NaCl, 1% (w/v) Nonidet P-40, 0.5% sodium deoxycholate, and 0.1% SDS) containing 1/100 (v/v) protease inhibitor cocktail (Cat number P8340, Sigma-Aldrich, St. Louis, MO, USA) and incubated at 4°C for 30 min with vortexing. After centrifugation at 14,000 ×g, at 4°C, for 30 minutes, supernatants were recovered as protein extracts. Aliquots (15 *μ*g for each) of protein samples were mixed with SDS sample buffer containing 2-mercaptoethanol, heated at 40°C for 15 minutes, cooled on ice, and then electrophoresed at 180 V in 10% polyacrylamide gel (Cat number 2331850, E-T15L, ATTO, Tokyo, Japan) for one hour. Gels were blotted on PVDF membranes (Cat number EH-2222, FluoroTrans W Membrane, Pall Corporation, Port Washington, NY, USA) at 1.0 mA/cm^2^ for one hour.

The membranes were incubated at room temperature for 30 minutes in blocking buffer (0.3% skim milk, 1% BSA in 0.05% Triton-TBS), hybridized with 0.5 *μ*g/mL of the anti-human rabbit polyclonal AQP1 antibody (AP23362PU-N, Acris Antibodies GmbH, Herford, Germany) or 1 *μ*g/mL of the anti-human rabbit polyclonal AQP3 antibody (Cat number ab125219, Abcam, Cambridge, UK) in 0.05% Triton-TBS containing 0.03% skim milk and 0.1% BSA at 4°C overnight, and washed three times with 0.1% TBST. The membranes were then incubated at room temperature for three hours in 0.05% Triton-TBS containing 0.03% skim milk, 0.1% BSA, and the HRP-linked anti-rabbit IgG antibody (1 : 20,000 diluted, Cat number 7074S, Cell Signaling Technology, Danvers, MA, USA) and washed three times with 0.1% TBST. Signals were detected using the SuperSignal West Pico Chemiluminescent Substrate (Cat number 34077, Thermo Scientific, Waltham, MA, USA).

After exposure to X-ray films, the membranes were incubated in 0.1 M glycine-HCl (pH 2.0) at room temperature for 30 minutes to remove the antibodies. GAPDH signals as loading controls were detected as described above using 0.1 *μ*g/mL of the anti-GAPDH mouse monoclonal antibody (Cat number AM4300, Ambion, Austin, TX, USA) as the primary antibody and HRP-linked anti-mouse IgG antibody (1 : 20,000 diluted, Cat number 7076S, Cell Signaling Technology, Danvers, MA, USA) as the secondary antibody. Densitometric analysis of Western blotting signals was performed using image-analyzing software. AQP1 signal values divided by those of the corresponding GAPDH signals (AQP1/GAPDH) were statistically evaluated.

## 3. Results

**Figure 1:**
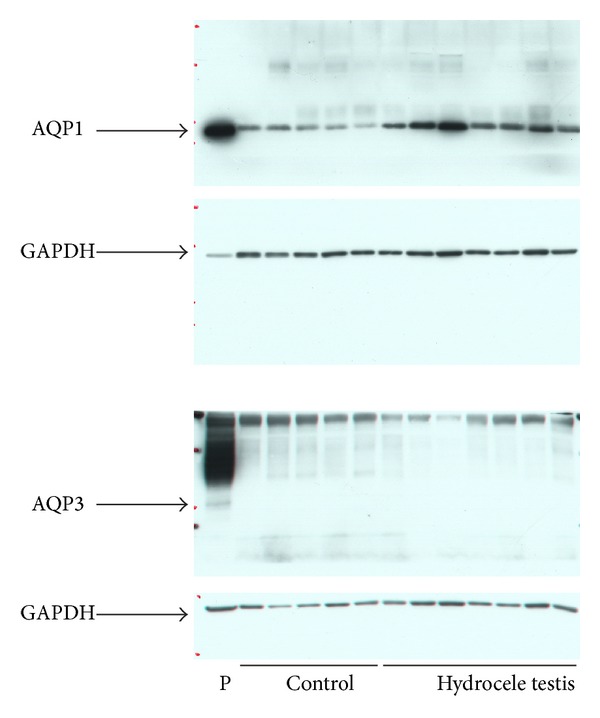
Expression of AQP1 and AQP3 proteins in the tunica vaginalis. P: human kidney tissue as a positive control for AQP1 and AQP3; ages of hydrocele patients: 57–84 years (median: 73); ages of control patients: 75–82 years (median: 78).

**Figure 2:**
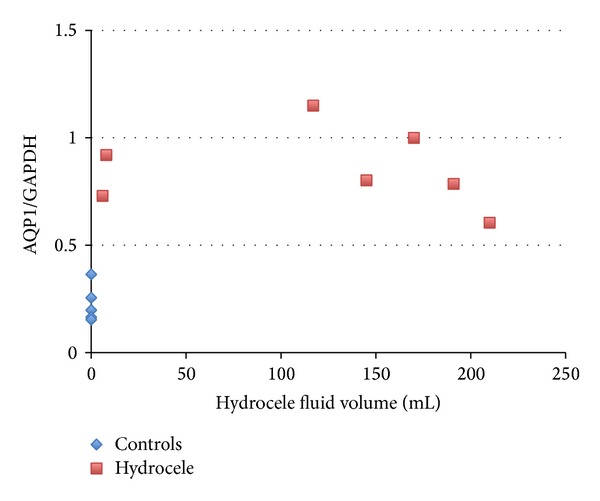
Scatter diagram of the ratio of the AQP1 signal to GAPDH and volume of hydrocele fluid. Controls were plotted with a fluid volume of zero.

Western blotting: AQP1 protein expression in the tunica vaginalis (Figure 1) was significantly higher in patients with adult-onset hydrocele testis (*n* = 7) than in the controls (*n* = 5). AQP1/GAPDH ratios were 0.897 ± 0.158 versus 0.234 ± 0.098, respectively (*P* < 0.001 by an unpaired* t*-test). As shown in a scatter diagram (Figure 2), the expression of AQP1/GAPDH was similar between the two cases with hydrocele fluid of less than 10 mL and other cases with more than 100 mL. The AQP3 protein was not detected in the tunica vaginalis of hydrocele patients or controls.

**Figure 3:**
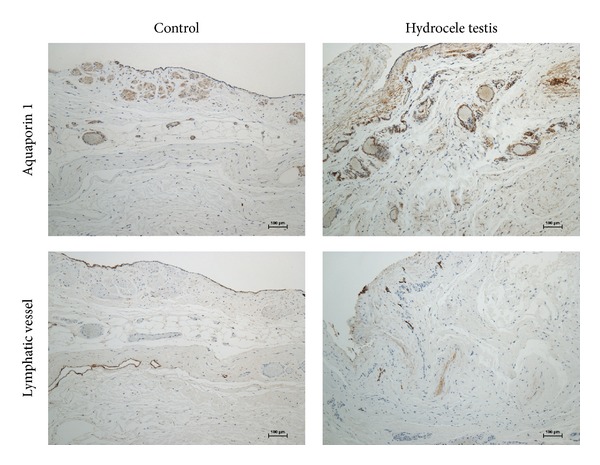
Histochemistry of aquaporin 1 and lymphatic vessels.

**Figure 4:**
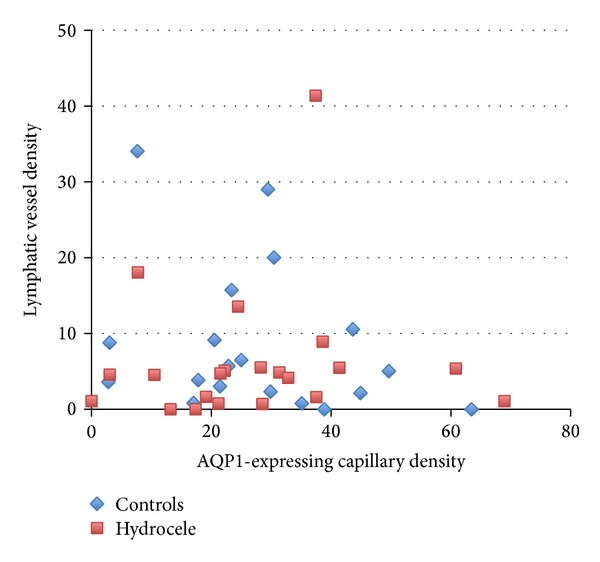
Scatter diagram of AQP1-expressing capillary density (counts/mm^2^) and lymphatic vessel density (counts/mm^2^).

Morphometric analysis of AQP1-expressing capillaries and lymphatic vessels in the tunica vaginalis was as follows. Histochemically, AQP1 expression in the tunica vaginalis was localized in vascular endothelial and smooth muscle cells (Figure 3). No significant differences were observed in the density of AQP1-expressing capillaries in the tunica vaginalis between the controls and hydrocele patients (27.6 ± 16.2 versus 28.2 ± 16.7/mm^2^, resp.; mean ± SD; *P* = 0.92 by an unpaired* t*-test). Lymphatic vessel densities were also comparable (8.8 ± 9.9 versus 6.4 ± 9.4/mm^2^, resp.; *P* = 0.45 by an unpaired* t*-test). Hydrocele testis specimens and controls were indistinguishable from each other on a scatter diagram showing the AQP1-expressing capillary density and lymphatic vessel density (Figure 4).

Biochemical analysis of the serum and hydrocele fluid was as follows. No significant differences were observed in the osmolality or potassium, chloride, glucose, urea nitrogen, creatinine, or calcium measurements between the serum and hydrocele fluid, as shown in Table 1. Sodium levels were higher in the hydrocele fluid than in the serum. The mean of the total protein was lower in the hydrocele fluid than in the serum, but it was not significantly different based on an unpaired* t*-test due to the very wide range of protein values in the hydrocele fluid (0.1 to 8.7 g/dL, median: 4.7 g/dL).

## 4. Discussion

The pathogenesis of adult-onset primary noncommunicating hydrocele testis remains unclear. Our findings suggest the involvement of the overexpression of the AQP1 protein in individual capillary endothelial cells in the tunica vaginalis in the development of hydrocele testis, because the level of expression of the AQP1 protein in the tunica vaginalis is higher in patients with primary hydrocele than in controls, while the densities of AQP1-expressing capillaries are similar between them. The upregulation of capillary AQP1 allows water to flow from the blood through the interstitium and into the tunica vaginalis layers across the mesothelium. Water transported between these layers is absorbed from lymphatic vessels in the tunica vaginalis. Hydrocele fluid may accumulate under conditions in which enhanced water efflux from capillaries due to an increase in their expression of AQP1 exceeds the maximum capacity for lymphatic fluid absorption.

Following water accumulation, the tunica vaginalis functions as a dialysis membrane and the equilibration of substances occurs, as in peritoneal dialysis. Therefore, the biochemical concentrations of most substances, except for sodium and total protein, are similar between the serum and hydrocele fluid, as shown in Table 1, resulting in their identical osmolalities. The osmolality of hydrocele fluid, as well as that of serum, may be generally calculated using the formula: 2[Na+] + [Glucose]/18 + [BUN]/2.8. As shown in Table 1, the glucose concentration in hydrocele fluid is relatively lower and more constant compared with that in serum and not influenced by the wider distribution of serum glucose levels for unknown reasons. When the osmolalities of the serum and hydrocele fluid are similar, the sodium concentration may compensate for the lower levels of glucose in hydrocele fluid. Therefore, sodium levels may be significantly higher in hydrocele fluid than in serum. Hoshino and colleagues previously analyzed biochemical components in the fluid of adult-onset hydrocele [13]. In that report, sodium, potassium, and urea nitrogen concentrations in hydrocele fluid were similar to those in the serum. Total protein concentrations in hydrocele fluid vary very widely from nearly zero to as high as those in the serum. This may be because the permeability of capillaries with large pores, with a radius of 200–300 A, that transport macromolecules as proteins [1], varies depending on each individual tunica vaginalis. In addition, the variable rates of lymphatic drainage of protein in the tunica vaginalis may lead to the wider distribution of total protein levels in the hydrocele fluid.

The densities of lymphatic vessels in the tunica vaginalis were similar between patients with adult-onset hydrocele and the controls. Whether or not fluid absorption from lymphatic vessels is similar remains to be determined. The ability of lymphatic vessels to absorb fluid is difficult to quantify. The scatter diagram showing the densities of AQP1-expressing capillaries and lymphatic vessels could not be used to differentiate between patients with hydrocele testis and the controls. Nevertheless, total fluid absorption from lymphatic vessels may be greater in individuals who have no or little hydrocele fluid. Hydrocele fluid may accumulate in the secondary hydrocele in the presence of scrotal inflammation, cancer, or trauma due to a reduction in the ability of lymphatic vessels to absorb fluid. The balance between water efflux from capillaries and the absorption of fluid into lymphatic vessels must be significantly disrupted in order for noncommunicating hydrocele testis to develop.

The reason why the AQP1 protein is upregulated in the tunica vaginalis of patients with adult-onset primary hydrocele remains unknown. Epigenetics such as DNA methylation and single nucleotide polymorphism in the AQP1 gene may be involved in the pathogenesis of adult-onset hydrocele testis. AQP1 gene polymorphism was assessed in primary open-angle glaucoma [14], and hypomethylation of the AQP1 gene was indicated in salivary gland adenoid cystic carcinoma [15]. In a previous study, AQP3 was detected in human peritoneal tissue by the reverse transcription-polymerase chain reaction (RT-PCR) method [12], which is very sensitive, while it was not detected in the tunica vaginalis by the less sensitive Western blotting analysis used in the present study. The contribution of AQP3 to the transportation of water in the tunica vaginalis appears to be negligible.

## 5. Conclusions

Overexpression of the AQP1 protein in the tunica vaginalis may contribute to the development of adult-onset primary hydrocele testis as another aquaporin disease.

## Figures and Tables

**Table 1:** Biochemical analysis of the serum and hydrocele fluid.

	Osmolality	TP	Na*	K	Cl	Glucose	UN	Cre	Ca
Serum	290 ± 4	7.3 ± 0.3	139 ± 2	4.1 ± 0.6	103 ± 3	131 ± 46	17 ± 9	0.90 ± 0.10	9.2 ± 0.6
Hydrocele fluid	289 ± 6	4.7 ± 3.3	144 ± 2	3.7 ± 0.5	109 ± 6	103 ± 10	14 ± 2	0.87 ± 0.15	8.2 ± 1.6

*P = 0.002 by unpaired *t*-test, *n* = 7.
